# Prediction of Locoregional Recurrence-Free Survival of Oesophageal Squamous Cell Carcinoma After Chemoradiotherapy Based on an Enhanced CT-Based Radiomics Model

**DOI:** 10.3389/fonc.2021.739933

**Published:** 2021-09-24

**Authors:** Jie Kong, Shuchai Zhu, Gaofeng Shi, Zhikun Liu, Jun Zhang, Jialiang Ren

**Affiliations:** ^1^ Department of Radiation Oncology, The Fourth Hospital of Hebei Medical University, Shijiazhuang, China; ^2^ Department of Computed Tomography and Magnetic Resonance, The Fourth Hospital of Hebei Medical University, Shijiazhuang, China; ^3^ Pharmaceutical Diagnosis, GE Healthcare, Beijing, China

**Keywords:** oesophageal squamous cell carcinoma, chemoradiotherapy, radiomics, enhanced CT, locoregional recurrence-free survival

## Abstract

**Background and Purpose:**

Chemoradiotherapy is the standard treatment for moderate and advanced oesophageal cancer. The aim of this study was to establish a predictive model based on enhanced computed tomography examination, and to evaluate its clinical value for detecting locoregional recurrence-free survival (LRFS) in cases of oesophageal squamous cell carcinoma after radiotherapy.

**Materials and Methods:**

In total, 218 patients with pathologically diagnosed oesophageal squamous cell carcinoma who received radical chemoradiotherapy from July 2016 to December 2017 were collected in this study. Patients were randomly divided into either a training group (n=153) or a validation group (n=65) in a 7:3 ratio. Clinical patient information was then recorded. The enhanced computed tomography scan images of the patients were imported into 3D-slicer software (version 4.8.1), and the radiomic features were extracted by the Python programme package. In the training group, the dimensionality reduction of the radiomic features was implemented by Lasso regression, and then a radiological label, the model of predicting LRFS, was established and evaluated. To achieve a better prediction performance, the radiological label was combined with clinical risk factor information to construct a radiomics nomogram. A receiver operating characteristic curve was used to evaluate the efficacy of different models. Calibration curves were used to assess the consistency between the predicted and observed recurrence risk, and the Hosmer-Lemeshow method was used to test model fitness. The C-index evaluated the discriminating ability of the prediction model. Decision curve analysis was used to determine the clinical value of the constructed prediction model.

**Results:**

Of the 218 patients followed up in this study, 44 patients (28.8%) in the training group and 21 patients (32.3%) in the validation group experienced recurrence. There was no difference in LRFS between the two groups (*χ^2 =^* 0.525, P=0.405). Lasso regression was used in the training group to select six significant radiomic features. The radiological label established using these six features had a satisfactory prediction performance. The C-index was 0.716 (95% CI: 0.645–0.787) in the training group and 0.718 (95% CI: 0.612–0.825) in the validation group. The radiomics nomogram, which included the radiological label and clinical risk factors, achieved a better prediction than the radiological label alone. The C-index was 0.742 (95% CI: 0.674–0.810) in the training group and 0.715 (95% CI: 0.609–0.820) in the validation group. The results of the calibration curve and decision curve analyses indicated that the radiomics nomogram was superior in predicting LRFS of oesophageal carcinoma after radiotherapy.

**Conclusions:**

A radiological label was successfully established to predict the LRFS of oesophageal squamous cell carcinoma after radiotherapy. The radiomics nomogram was complementary to the clinical prognostic features and could improve the prediction of the LRFS after radiotherapy for oesophageal cancer.

## Introduction

Oesophageal squamous cell carcinoma (ESCC) is the major histological subtype of oesophageal cancer, especially in high-incidence areas such as China (1–3). Most patients diagnosed with locally advanced oesophageal cancers lose the opportunity for surgery at the time of diagnosis; instead, the standard treatment of concurrent chemoradiotherapy is recommended (4). However, local recurrence is still the main cause of treatment failure (5). Once recurrence occurs, the patient’s prognosis is usually poor, with a reported survival time of 3–10 months (6, 7). Reducing the recurrence of oesophageal cancer is an urgent problem to be solved in clinical practice (8). For patients receiving concurrent chemoradiotherapy, the clinical stage is the most important factor for prognosis. However, due to tumour heterogeneity, large differences in recurrence occur even among patients at the same stage (9). Accurate quantification of the inherent heterogeneity of oesophageal cancer and a search for the factors affecting recurrence are crucial to improve local control and prolong survival (10). This will allow clinicians to adopt more active treatments (radiotherapy combined with targeted therapy, immunotherapy, etc.) in high-risk patients.

Radiomics technology is a great innovation in the field of medical image analysis (11). It extracts quantitative features from computed tomography (CT), magnetic resonance, and positron emission tomography images, among other modalities, to locate the changes in the internal characteristics of a tumour. The technology then combines these changes with the biological behaviour of the tumours to reach a precise diagnosis and treatment. Moreover, many studies have focused on predicting therapeutic efficacy (12–15), but few have investigated the prediction of locoregional recurrence-free survival (LRFS) in patients post-radiotherapy.

To explore the relationship between clinical characteristics, radiomic features, and local control after radiotherapy, we performed an enhanced CT scan before and after radiotherapy to select the factors with clinical value. We then constructed a model based on the radiomic characteristics combined with the clinical parameters. The model was used to predict the LRFS of ESCC patients receiving radiotherapy and to provide a theoretical reference for treatment.

## Materials and Methods

### Patients

Patients with pathologically confirmed ESCC and a Karnofsky Performance Status ≥70 who were receiving three-dimensional conformal or intensity-modulated radiotherapy were included in this study. Patients were excluded if they met the following criteria: 1) distant metastasis; 2) low-dose palliative radiotherapy; 3) preoperative or postoperative adjuvant radiotherapy; 4) incomplete clinicopathological information; 5) oesophageal fistula and oesophageal stent implantation; 6) image artifacts or tumour volumes are too small to be recognised on CT images, resulting in poor visualisation quality; or 7) previous malignant tumour history.

Ultimately, 218 patients who received three-dimensional conformal or intensity-modulated radiotherapy at the Fourth Hospital of Hebei Medical University from July 2016 to December 2017 were enrolled. The median age was 67 years (37–84 years) and the median lesion length was 5.0 cm (1.0–12.0 cm). All patients received electronic gastroscopy, oesophageal barium meal contrast, chest enhanced CT scan and abdominal ultrasound or CT examination before treatment, according to the eighth edition of the American Joint Committee on Cancer staging criteria (16).

### Radiotherapy

Gross tumour volume included the primary oesophageal tumour and regional lymph nodes. The criteria for determining oesophageal lesions on CT images were oesophageal wall thickness >5 mm or non-airless oesophagus diameter >10 mm, localised or whole oesophageal wall thickening, and/or local lumen stenosis. The clinical target volume (CTV) was obtained by expanding the GTV to a margin to 2.0-3.0 cm at the long axis and 0.5 cm at the lateral axis. The planning target volume (PTV) was reached by CTV plus a margin of 0.5 cm. The prescription dose for the whole group was 50.0–66.0 Gy, the median dose was 60.0 Gy, and a single dose was 1.8–2.2 Gy.

### Chemotherapy

A total of 90 patients received 1–2 cycles of concurrent chemotherapy, with the main regimens of FP (cisplatin, 12.5 mg/m^2^×5 days or 25 mg/m^2^×3 days; 5-fluorouracil, 450 to 500 mg/m^2^×5 days) or TP (paclitaxel, 135 mg/m^2^, d1,8 days; cisplatin, 25 mg/m^2^, d2, 3, 4 days, 28 days as a cycle, then 1, 5 weeks of administration).

### CT Image Acquisition

CT images were collected before and within 1 month after chemoradiotherapy. All patients underwent standard chest contrast-enhanced CT scanning with a CT scanner (SOMATOM Definition Flash CT, SOMATOM Sensation Open CT, Forchheim, Germany). Scan parameters were as follows: tube voltage, 120 kV; tube current, 110 mA; scanning matrix, 512 x 512; conventional scanning layer thickness, 5.0 mm; reconstruction layer thickness, 1.0 mm; mediastinal window width, 350 HU; window position, 40 HU; lung window width, 1200 HU; and window position, -600 HU. In this study, enhanced CT images were used for tumour delineation and feature extraction.

### CT Image Segmentation

The ROI profiling process is shown in **Supplementary Figure 1**, Arterial-phase CT images of 218 patients which were retrieved from PACS(Carestream) were imported into the 3D Slicer software (version 4.8.1, http://www.slicer.org), The tumours were manually segmented slice by slice using the software. and an attending physician with more than 5 years of clinical experience independently outlined the region of interest (ROI) of the oesophageal primary tumours. The lesion was considered to be tumours when the oesophageal wall showed focal thickening of ≥5 mm on imaging. Intraluminal air and contrast agent, fatty tissues, tumour necrosis surrounding the lesion, and blood vessels near the gross tumour were removed from the ROI, defined as an area with attenuation values below -50 HU and over 300 HU. The attending physician sketched all tumour ROIs, and the associate chief physician randomly selected 40 cases of sketched tumour ROIs for a consistency test.

### Radiomic Feature Extraction and Selection

Radiomic features of the segmented 3D images were extracted using the Python programme package Pyradiomics 1.2.0.(Amsterdam Netherlands). A total of seven categories of imaging features were collected in this study. This included 18 first-order, 14 shape-based histogram, 24 grey level co-occurrence matrix, 16 grey level size zone matrix, 16 grey level run length matrix, 14 grayscale dependence matrix, and 5 neighbourhood grey-tone difference matrix features (17).

The intergroup correlation coefficient (ICC) was used to analyse the consistency of the radiomic features extracted from the ROI of the tumours in the training group. The features with good reproducibility (ICC>0.75) were selected. First, Spearman correlation analysis was performed for any two feature columns. R>0.9 indicated that the two features were highly correlated, and the features with large correlation coefficients with LRFS were retained. Second, the most useful predictive features were selected using the least absolute shrinkage and selection operator (LASSO) Cox regression model, which was applied to reduce high-dimensional data. Ten-fold cross-validation was used in the parameter tuning phase of the LASSO algorithm to extract the effective and predictive features.

### Construction of the Radiological Label (Rad-Score) and Radiomics Nomogram

After the imaging features were screened by means of dimensionality reduction, Cox regression was used to calculate the regression coefficient (β). The weighted linear formula was as follows:


rad−score=β0+β1x1+β2x2+β3x3+………+βnXn.

For a better prediction effect, a multivariate Cox proportional hazards model was utilised to build a radiomics nomogram. The nomogram was constructed by combining the radiomic signature with the mentioned conventional clinical parameters to determine the model with the optimal predictive performance.

### Validation of the Radiological Label (Rad-Score) and Radiomics Nomogram

The efficacy of the radiological label in predicting post-radiotherapy LRFS was determined using the receiver operating characteristic curve. The C-index was used to evaluate the discrimination power, which was defined as the agreement between the predicted and actual RFS probabilities; decision curve analysis was then used to determine the clinical value of the constructed prediction model. Higher clinical utility was observed the farther away the decision curve was from the two extreme curves (treat-all and treat-none). In addition, we used the calibration curve to assess the predictive accuracy and the agreement between the actual and predicted RFS. The Akaike information criterion (AIC) was used to assess the probability of overfitting; a smaller AIC value indicated a better fit of the model.

Moreover, we evaluated the association between the radiological label and LRFS of oesophageal cancer by using Kaplan-Meier analysis in the training group. The optimal cut-off point was calculated to maximise the selection of rank statistics using X-tile software(New Haven USA). Then, the patients with radiological label values above the cut-off point were allocated into a high-risk group, while those with values below the cut-off point were allocated into the low-risk group. The log-rank test was used to measure the difference in survival curves between the two groups. We then performed the same analysis for the radiomics nomogram.

### Statistical Analysis

Statistical analysis was performed using R software (version 3.4.4) and SPSS version 25.0 (IBM). Comparisons of patient characteristics were performed using the Mann-Whitney U-test or two-sample t-test. Univariate analysis used the Kaplan-Meier method to calculate and compare the LRFS of different groups of oesophageal cancer patients. A Cox proportional hazards model was utilised to screen the independent influencing factors of LRFS. P<0.05 was statistically significant.

## Results

### Analysis of the General Clinical Characteristics of Patients

In total, 218 patients (149 males, 69 females) with ESCC were enrolled in the study, with a median age of 67.0 (37.0–84.0) years. Patients were divided into either training or validation groups in a 7:3 ratio. There were 153 patients in the training group, including 106 males and 47 females, with a median age of 67.0 (37.0–81.0) years. The validation group consisted of 65 patients, including 43 males and 22 females, with a median age of 69.0 (46.0–84.0) years. There were no differences in the clinical characteristics between the two groups; P-values ranged from 0.051 to 0.982 (**Table 1**).

**Table 1:** The distribution of general clinical factors in the training cohort and validation cohort.

Factors	Training cohort (n = 153)	Validation cohort (n = 65)	X^2^/t	P
Age (years)	65.98 ± 8.73	67.52 ± 8.41	1.206	0.229
Gender				
Male	105	44	0.018	0.982
Female	48	21		
Location				
Cervical	11	0	5.349	0.148
Upper	41	16		
Middle	77	37		
Lower	24	12		
Length (cm)	5.35 ± 1.95	5.29 ± 2.70	0.211	0.833
Maximum tumour wall thicknesses (Pre-RT) (cm)	1.43 ± 0.47	1.45 ± 0.32	0.377	0.706
Maximum tumour wall thicknesses (Post-RT) (cm)	1.09 ± 0.43	1.45 ± 0.36	1.065	0.288
T stage				
T1-3	118	44	2.126	0.145
T4	35	21		
Tracheal invasion				
No	121	49	0.364	0.546
Yes	32	16		
Prevertebral soft tissue invasion				
No	144	58	1.602	0.206
Yes	9	7		
Aortic invasion				
No	144	56	3.819	0.051
Yes	9	9		
Supraclavicular lymph node metastasis				
Yes	32	14	0.011	0.918
No	121	51		
N stage				
N0	14	7	1.727	0.631
N1	46	24		
N2	63	21		
N3	30	13		
TNM stage				
I-III	97	37	0.808	0.369
IVa	56	28		
Chemotherapy				
Yes	94	38	0.169	0.681
No	59	27		
Chemoradiotherapy				
Yes	67	23	1.330	0.249
No	86	42		
Radiation dose (Gy)				
<60	53	22	1.707	0.426
60	49	16		
>60	51	27		
Short-term clinical effect				
CR	44	18	0.025	0.873
Non-CR	109	47		

### Locoregional Recurrence-Free Survival of Patients

All patients with oesophageal cancer were followed up for the full length of the follow-up period. By the end of the last follow-up visit, 44 patients (28.8%) in the training group and 21 patients (32.3%) in the validation group had recurrence. The LRFS of patients in the training group at 1, 2 and 3 years was 77.8%, 71.2%, and 71.2%, and that of patients in the validation group was 75.4%, 66.2%, and 66.2%, respectively. There was no statistically significant difference in LRFS between the two groups (*χ^2^ = *0.525, P=0.405). Univariate analysis of the data in the training group showed that the length of the lesion, the maximum layer wall thickness of the lesion before and after radiotherapy, aortic invasion, TNM stage, and the short-term efficacy after radiotherapy were correlated with LRFS after radiotherapy (P<0.05). The detailed results are listed in **Table 2**.

**Table 2:** Relationship between general clinical characteristics and LRFS in patients with oesophageal cancer.

Factors	Training cohort LRFS (%)			P	Validation cohort LRFS (%)			P
	(n = 153)				(n = 65)			
	1-year	2-year	3-year		1-year	2-year	3-year	
Gender								
Male	75.2	69.5	69.5	0.423	71.4	71.4	71.4	0.649
Female	83.3	75.0	75.0		77.3	68.2	63.6	
Location								
Cervical	63.6	63.6	63.6	0.401	–	–	–	0.140
Upper	87.8	78.0	78.0		93.5	87.5	87.5	
Middle	72.7	66.2	66.2		73.2	64.9	59.5	
Lower	83.3	79.2	79.2		58.3	58.3	58.3	
Lesion length								
≤5 cm	85.9	77.6	77.6	0.035	78.4	73.0	73.0	0.235
>5 cm	70.6	63.2	63.2		71.4	60.7	57.1	
Maximum tumour wall thicknesses (Pre-RT)								
≤1.5 cm	84.4	76.7	76.7	0.042	79.5	69.2	64.1	0.724
>1.5 cm	68.3	63.5	63.5		69.2	69.2	69.2	
Maximum tumour wall thicknesses (Post-RT)								
≤1.5 cm	80.7	73.3	73.3	0.048	79.2	71.7	71.7	0.540
>1.5 cm	55.6	55.6	55.6		60.0	60.0	60.0	
T stage								
T1-3	80.5	74.6	74.6	0.099	79.5	72.7	68.2	0.449
T4	68.6	60	60		66.7	61.9	61.9	
Tracheal invasion								
No	79.3	73.6	73.6	0.267	77.6	71.4	67.3	0.533
Yes	71.9	62.5	62.5		68.8	62.5	62.5	
Prevertebral soft tissue invasion								
No	77.8	70.8	70.8	0.691	85.7	71.4	71.4	0.784
Yes	77.8	77.8	77.8		74.1	69	65.5	
Aortic invasion								
No	79.9	72.9	72.9	0.032	76.8	69.6	66.1	0.938
Yes	44.4	44.4	44.4		66.7	66.7	66.7	
Supraclavicular lymph node metastasis								
Yes	74.4	70.1	70.1	0.537	70.8	62.5	58.3	0.036
No	88.6	74.3	74.3		88.2	88.2	88.2	
N stage								
N0	85.7	85.7	85.7	0.135	71.4	42.9	28.6	0.093
N1	80.4	73.9	73.9		70.8	62.5	62.5	
N2	81	73	73		71.4	71.4	71.4	
N3	63.3	56.7	56.7		92.3	84.6	94.6	
TNM stage								
I-III	83.5	77.3	77.3	0.025	78.4	70.3	67.6	0.606
IVa	67.9	60.7	60.7		71.4	67.9	64.3	
Albumin level (Pre-RT) (g/L)								
High (≥40)	72.4	67.1	67.1	0.259	69.7	63.6	60.6	0.333
Low (<40)	82.7	74.7	74.7		80	73.3	73.3	
Chemotherapy								
Yes	79.7	72.9	72.9	0.728	73.7	71.1	68.4	0.668
No	76.6	70.2	70.2		77.8	66.7	63	
Chemoradiotherapy								
Yes	79.1	73.3	73.3	0.573	65.2	60.9	60.9	0.408
No	76.1	68.7	68.7		81	73.8	69	
Radiation dose(Gy)								
<60	77.4	75.5	75.5	0.512	68.2	63.6	59.1	0.401
60	81.6	73.5	73.5		62.5	62.5	62.5	
>60	78.4	64.7	64.7		88.9	77.8	74.1	
Short-term clinical effect								
CR	88.6	86.4	86.4	0.01	88.9	83.3	77.8	0.194
Non-CR	73.4	65.1	65.1		70.2	63.8	61.7	

### Selection of Radiomic Features Associated With Local Recurrence of Oesophageal Carcinoma

**Figure 1:**
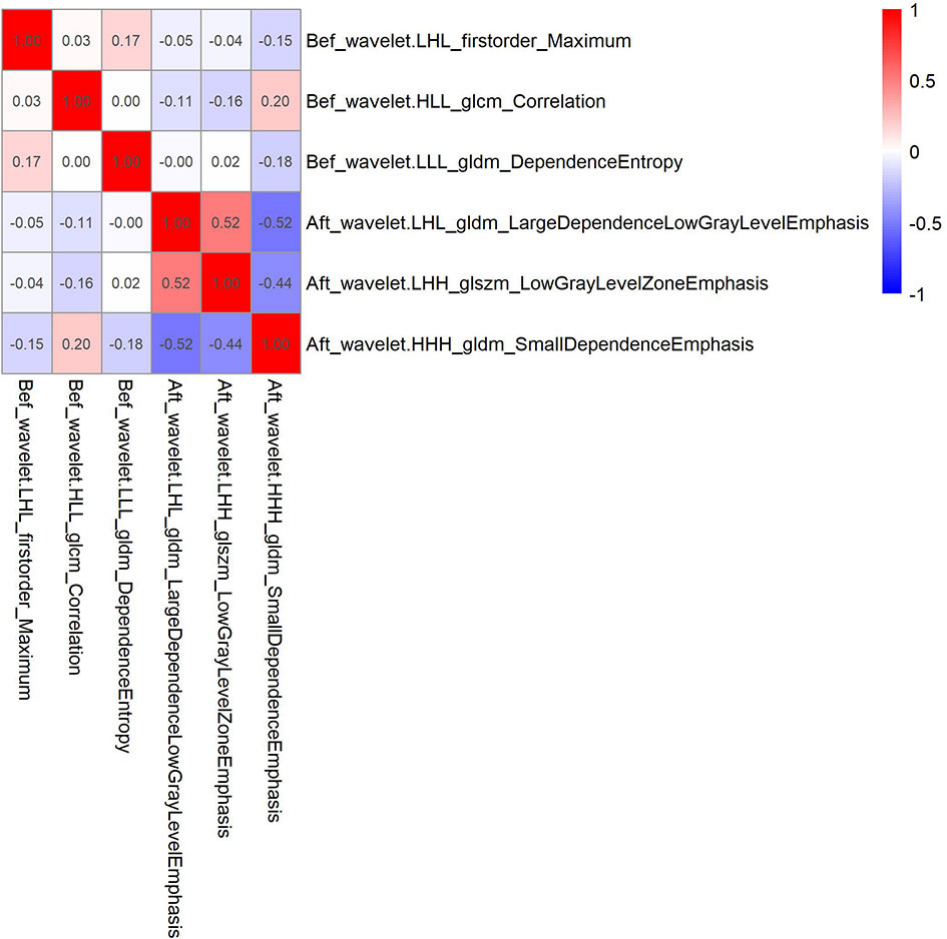
Correlation coefficient between 6 radiomics features screened by Lasso regression in the training cohort.

Of the 1037 radiomic features extracted from the CT images, 654 had an ICC value>0.75, indicating high reproducibility. Spearman correlation analysis was used to remove features with correlation coefficients greater than 0.9. Basing on the LASSO Cox regression model for LRFS, six radiomic features, including three before and three after radiotherapy, were selected. The correlation coefficients between the screened radiomics features are shown in **Figure 1**.

### Construction and Validation of the Radiological Label

A radiological label was established to predict LRFS in patients with oesophageal cancer after radiotherapy. The formula was expressed as follows:


$$Rad-score=-0.506*Bef_{wavelet}\cdot LHL_{firstorder_{maximum}}-0.338*Bef_{wavelet}\cdot HLL_{glcm_{Correlation}}+0.213*Bef_{wavelet}\cdot LLL_{gldm_{DependenceEntropy}}-0.621*Aft_{wavelet}\cdot LHL_{gldm_{L\arg eDependenceLowGrayLevel^{Emphasis}}}-0.40*Aft\_wavelet\cdot LHH\_glszm\_LowGrayLevelZoneEmphasis-0.86*Aft\_wavelet\cdot HHH\_gldm\_SmallDependenceEmphasis$$

**Figure 2:**
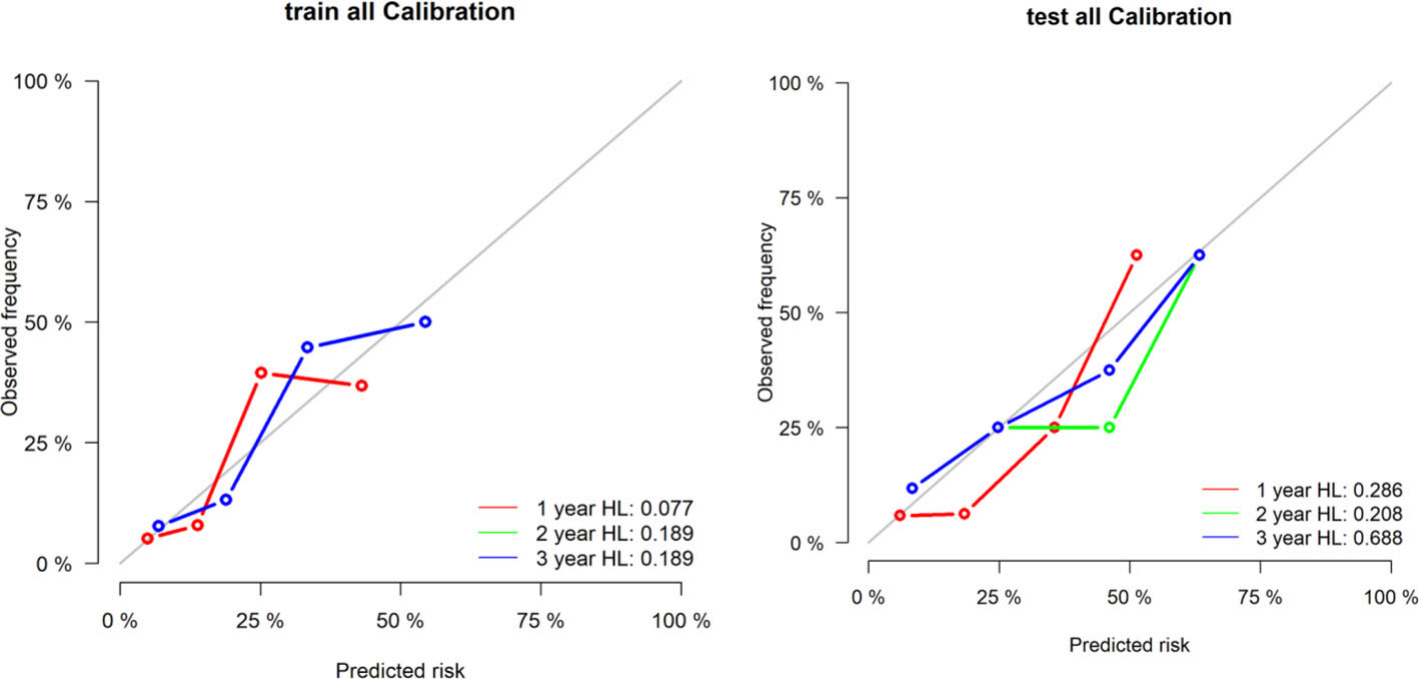
Calibration curves of the radiological label were plotted to assess the agreement between LRFS predicted by the model and the observed LRFS.

The area under the curve of the radiological label for predicting LRFS after radiotherapy was 0.767 (95% CI: 0.688–0.846) in the training group and 0.728 (95% CI: 0.601–0.856) in the validation group. The C-index of the radiological labels in the training and validation groups were 0.716 (95% CI: 0.645–0.787) and 0.718 (95% CI: 0.612–0.825), respectively. The results of the calibration curves suggest that the radiological labels had a high goodness of fit (P>0.05) (**Figure 2**).

**Figure 3:**
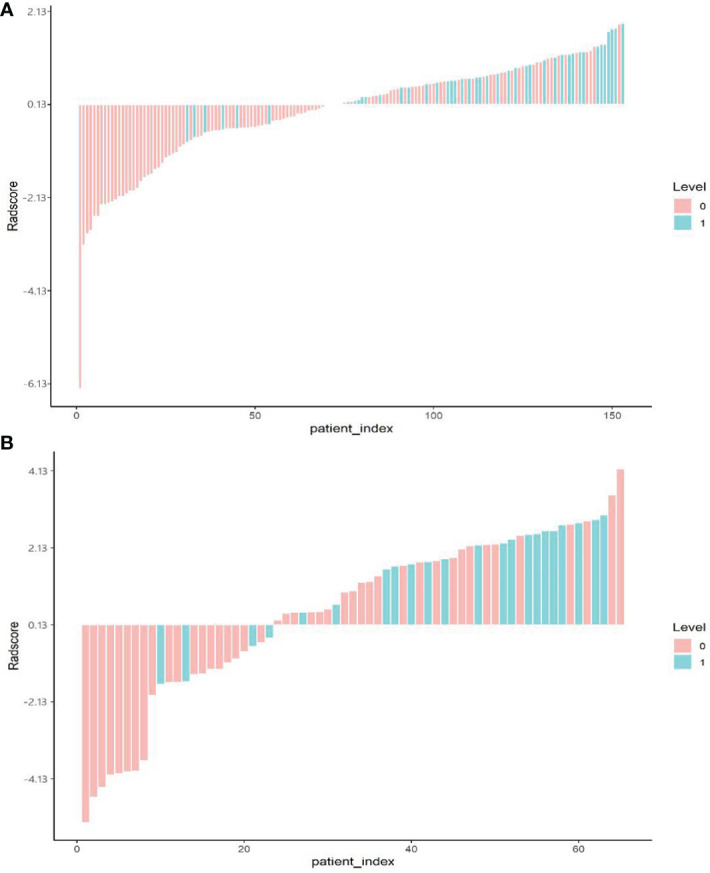
Bar plot of the radiomics signature value for each patient in the training cohort **(A)** and the validation cohort **(B).** 0, good control (red); 1, local uncontrolled or recurrent (green).

The optimal cut-off point of the radiological label was 0.13, as generated by the X-tile software. Patients were divided into two groups: patients with radiomics signature ≥0.13 were classified as the high-risk group, and those with a score <0.13 were classified as the low-risk group. The distributions of radiomic signature values calculated from the training and validation groups are presented in **Figure 3**.

**Figure 4:**
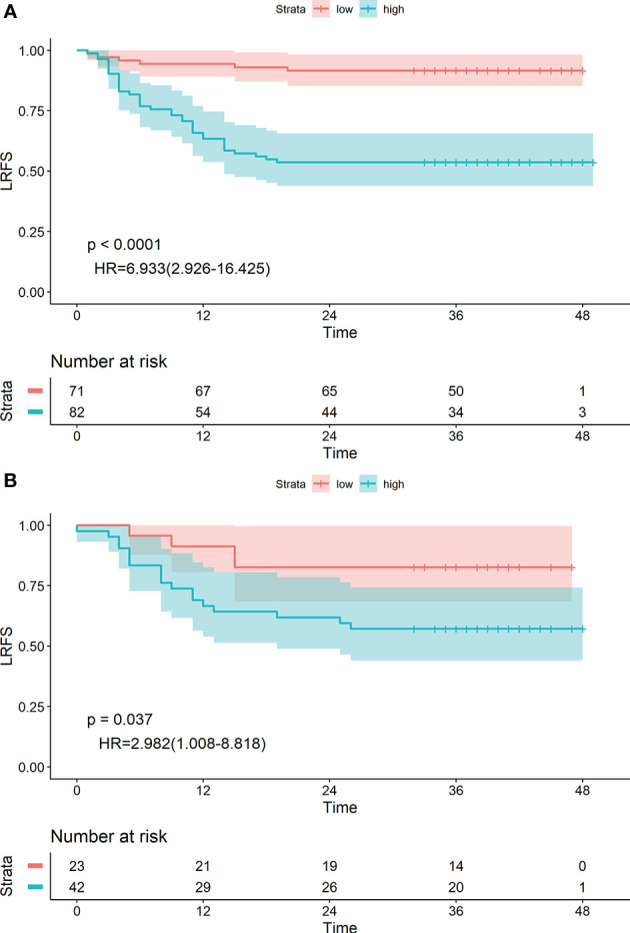
Kaplan-Meier survival analyses of high-risk and low-risk groups divided by radiological label in the training cohort **(A)** and validation cohort **(B)**.

The 1-, 2- and 3-year LRFS of patients in the low-risk group were 94.36%, 91.55%, and 91.55%, and those in the high-risk group were 65.85%, 53.66%, and 53.66%, respectively. The difference between groups was statistically significant (HR: 6.933 [2.926–16.425], P<0.0001) (**Figure 4A**). Similar results were obtained in the validation group (HR: 2.982 [1.006–8.818], P=0.037) (**Figure 4B**).

### Construction and Validation of the Radiomics Nomogram

Cox proportional hazard regression analysis showed that lesion length, TNM stage, and radiological label were independent predictors of LRFS after radiotherapy for oesophageal cancer. Based on the above results, we built a radiomics nomogram with the formula:


0.458∗lesion length+0.525∗TNM stage+0.946∗radiological label

**Figure 5:**
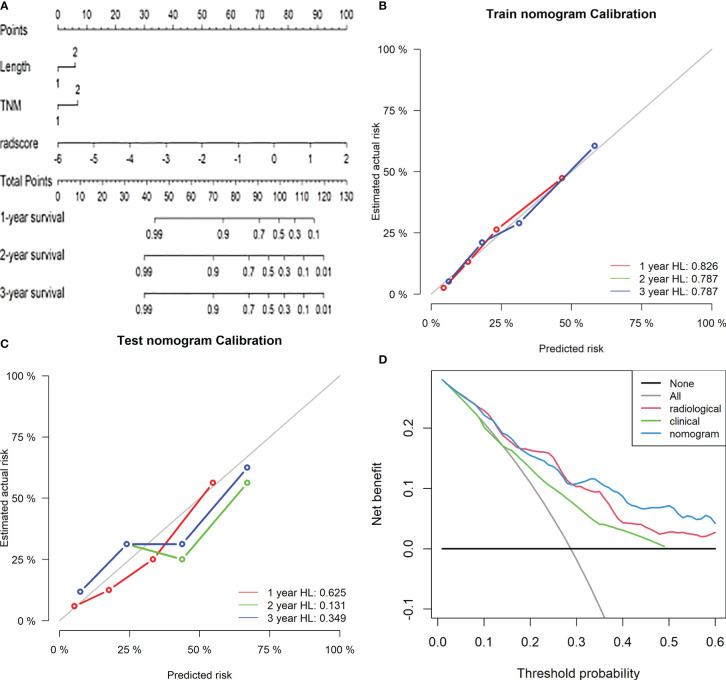
Radiomics nomogram for predicting LRFS of ESCC patients after radiotherapy. Length: 1, length of the oesophageal lesion ≤5 cm; 2, length of lesion >5 cm. TNM: 1, clinical stage I-III of oesophageal cancer; 2, clinical stage IV **(A)**. Calibration curves of the radiomics nomogram in the training **(B)** and validation **(C)** cohorts. Potential incremental values of the radiomics nomogram relative to the radiological label were evaluated by net reclassification improvement (NRI) **(D)**.

The area under the receiver operating characteristic curve was 0.790 (95% CI: 0.712–0.867) in the training group and 0.727 (95% CI: 0.598–0.856) in the validation group. The calibration curve suggested there was a high goodness of fit between the predictions of the radiomics nomogram and the actual probabilities of 1, 2, and 3-year LRFS (**Figure 5**). The C-index and AIC estimates for the different models are listed in **Table 3**. The C-index values of the radiomics nomogram were 0.742 (95% CI: 0.674–0.810) in the training cohort and 0.715 (95% CI: 0.609–0.820) in the validation cohort. Of all the models, this nomogram had the lowest AIC value in both the training group and the validation group. These results suggested that the predictive power of the radiomics nomogram for LRFS was significantly improved when compared with each feature set alone. Decision curve analysis showed that the radiomics nomogram produced a greater net benefit than the other predictive models, as presented in **Figure 5**.

**Table 3:** Discriminating performance of the radiological label and radiomics nomogram.

Model	AIC	Training cohort (n = 153)	Validation cohort (n = 65)
		C-index (95% CI)	C-index (95% CI)
Radiological label	410.78	0.716 (0.645–0.787)	0.718 (0.612–0.825)
Radiomics nomogram	399.28	0.742 (0.674–0.810)	0.715 (0.609–0.820)

**Figure 6:**
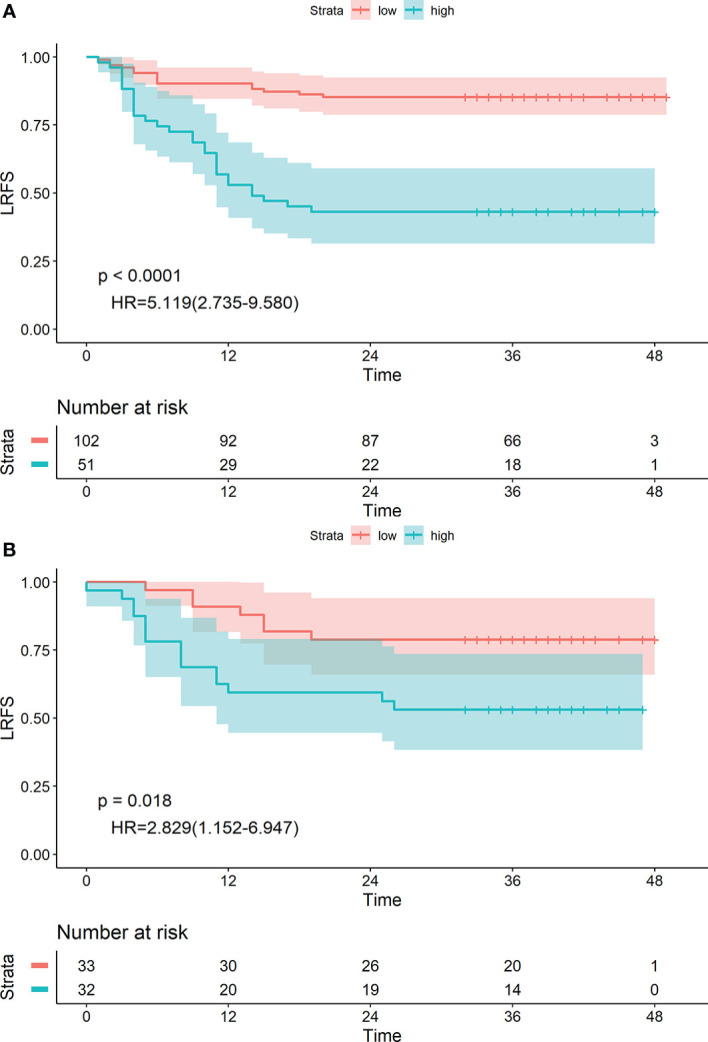
Kaplan-Meier survival analyses of high-risk and low-risk groups divided by the radiomics nomogram in the training **(A)** and validation **(B)** cohorts.

The radiomics nomogram yielded an optimal cut-off point of 0.56. Patients were divided into high-risk (≥0.56) and low-risk (<0.56) groups accordingly. The 1-, 2- and 3-year LRFS of patients in the low-risk group were 90.19%, 85.29%, and 85.29%, and those in the high-risk group were 56.86%, 43.14%, and 43.14%, respectively. The difference was statistically significant [HR: 5.119 (2.735–9.580), P<0.0001] (**Figure 6A**). Similar conclusions were reached in the validation group [HR: 2.829 (1.152–6.947), P=0.018] (**Figure 6B**).

## Discussion

As well known, chemoradiotherapy was the standard treatment for the patients of local advanced oesophageal cancer (12). However, a high local recurrence rate still remains (15), and once recurrence was diagnosed, the median OS was 1 year (18), Many studies have suggested that tumour size, degree of differentiation and lymphovascular invasion were the independent factors in predicting the recurrence of oesophageal cancer after treatment (6, 19, 20), but these studies mainly focused on patients who received surgical treatment, and were not applicable to patients receiving radical chemoradiotherapy. Therefore, there is a need to find more effective markers to predict the LRFS of oesophageal cancer after radiotherapy, as this can help us to identify patients with a high recurrence risk to take more active intervention measures.

In clinical practice, CT plays an important role in the diagnosis, staging, efficacy evaluation, and prognosis monitoring of oesophageal cancer (21). However, images obtained in clinical applications cannot capture the underlying tumour characteristics. The value of the our study is that it proposes a novel method for predicting LRFS of patients with oesophageal cancer after chemoradiotherapy. The results of the study showed that radiological label, a non-invasive, quantitative, and low-cost parameter, was an independent factor in predicting LRFS. we concluded that radiological label combined with clinical features can improve the predictive ability. This approach is expected to identify people at high risk of recurrence and support decision-making in clinical treatment for patients with oesophageal cancer.

In this study, we established a radiological label using enhanced CT images before and after radiotherapy for predicting LRFS in patients with oesophageal cancer. The model contains six radiomic features, five of which are textural features, which provide information about tumour heterogeneity for the assessment of the local oesophageal cancer recurrence. Many studies have explored the value of radiomics in clinical practice (22–28). The results of one study, carried out by Shen et al., suggested that by extracting preoperative CT image features, a radiomic signature can predict the lymph node metastasis status of oesophageal cancer. Thirteen radiomics features were significantly correlated with lymph node metastasis. Another study by Zhang et al. screened 11 imaging features that were significantly associated with the local control of nasopharyngeal carcinoma. Similarly, in our study, the radiological label that was established with six radiomics features obtained satisfactory results in predicting LRFS after radiotherapy for oesophageal cancer in both the training group (C-index, 0.716; 95% CI: 0.645–0.787) and validation group (C-index, 0.718; 95% CI: 0.612–0.825). The patients were stratified into high-risk and low-risk groups according to the radiological label value. The 1-, 2-, and 3-year LRFS values of the low-risk group were significantly higher than those of the high-risk group. The results of our study confirmed that the radiological label was an independent predictor of LRFS.

Our radiological label displayed a significant correlation with LRFS. However, clinical characteristics including lesion length, T stage, and N stage were also important influencing factors for local recurrence of oesophageal cancer (29), and these factors can be easily determined during treatment without increasing the burden on patients. Many studies have reported that combining radiological label with clinical risk factors can improve the accuracy of LRFS prediction (30–36). Therefore, we assumed that our model would achieve better performance when combined with these factors, and our results confirmed this hypothesis. The area under the receiver operating characteristic curve for our radiomics nomogram was 0.790 (95% CI: 0.712–0.867) in the training group and 0.727 (95% CI: 0.598–0.856) in the validation group. The C-index was 0.742 (95% CI: 0.674–0.810) in the training group and 0.715 (95% CI: 0.609–0.820) in the validation group. Encouragingly, our study was one of the first clinical studies to explore the use of radiomics in pretherapeutic assessment to predict LRFS after radiotherapy for oesophageal cancer.

Similarly, according to the principle of maximum selected rank statistics, the optimal cut-off point for predicting LRFS by radiomics nomogram was 0.56, and patients were divided into high-risk group and low-risk groups accordingly. There were significant differences in LRFS at 1-, 2-, and 3-year follow-up between the high-and low-risk groups based on this criterion, both in the training and validation sets. Our finding again supports the hypothesis that LRFS was better predicted in patients with oesophageal cancer after radiotherapy when the radiomics label was incorporated with clinical factors.

Admittedly, there were a few limitations in our study. First, patient representation was limited due to the study being conducted in a single-centre; however, as a regional radiotherapy centre, there were sufficient case resources. Furthermore, biases might have been present due to the retrospective nature of the study design; for example, the exact time of relapse of some patients was affected by memory bias over the follow-up period. Genomics was also an important method used to explore the heterogeneity of tumours and to implement individualised therapy. Therefore, to improve the accuracy of the prediction model, data from a larger multi-centre study is needed to further validate the stability and effectiveness of the model. In future clinical studies, key genetic markers that have been found to be closely related to local recurrence of oesophageal cancer should be added to the prediction model.

In conclusion, the predictive model established by combining enhanced-CT-derived radiomic characteristics with clinical risk factors has great potential and application prospect in predicting the local recurrence of oesophageal cancer after radiotherapy.

## Data Availability Statement

The datasets for this article are not publicly available because involving patient confidential information. Requests to access the datasets should be directed to the corresponding author.

## Ethics Statement

The studies involving human participants were reviewed and approved by Ethics committee of the Fourth Hospital of Hebei Medical University. The ethics committee waived the requirement of written informed consent for participation.

## Author Contributions

JK and SZ designed the study. JK and SZ accomplished the manuscript. JK collected the required CT data. ZL and JZ evaluated the curative effect of radiotherapy for oesophageal cancer and determined the local control status of oesophageal cancer. ZL collected and assembled the clinical data. JR performed the statistical analysis and interpretation. JK determined the selection of references and experimental standards. JK and ZL performed the data analysis and interpretation. SZ performed manuscript approval and modification. All authors contributed to the article and approved the submitted version.

## Funding

This study was supported by the National Natural Science Foundation of China (Grant No. 81872456) and the Health Innovation Special Fund of Hebei Province (Grant No. 213777104D).

## Conflict of Interest

JR was employed by GE Healthcare China.

The remaining authors declare that the research was conducted in the absence of any commercial or financial relationships that could be construed as a potential conflict of interest.

## Publisher’s Note

All claims expressed in this article are solely those of the authors and do not necessarily represent those of their affiliated organizations, or those of the publisher, the editors and the reviewers. Any product that may be evaluated in this article, or claim that may be made by its manufacturer, is not guaranteed or endorsed by the publisher.
