# Supplementary material for: Prediction of Locoregional Recurrence-Free Survival of Oesophageal Squamous Cell Carcinoma After Chemoradiotherapy Based on an Enhanced CT-Based Radiomics Model
Source: Front Oncol. 2021 Sep 24;11:739933. doi: 10.3389/fonc.2021.739933 (PMC8499696; doi:10.3389/fonc.2021.739933)
Supplement: Supplementary file 1 [file DataSheet_1.docx]

**C**

**C**

**B**


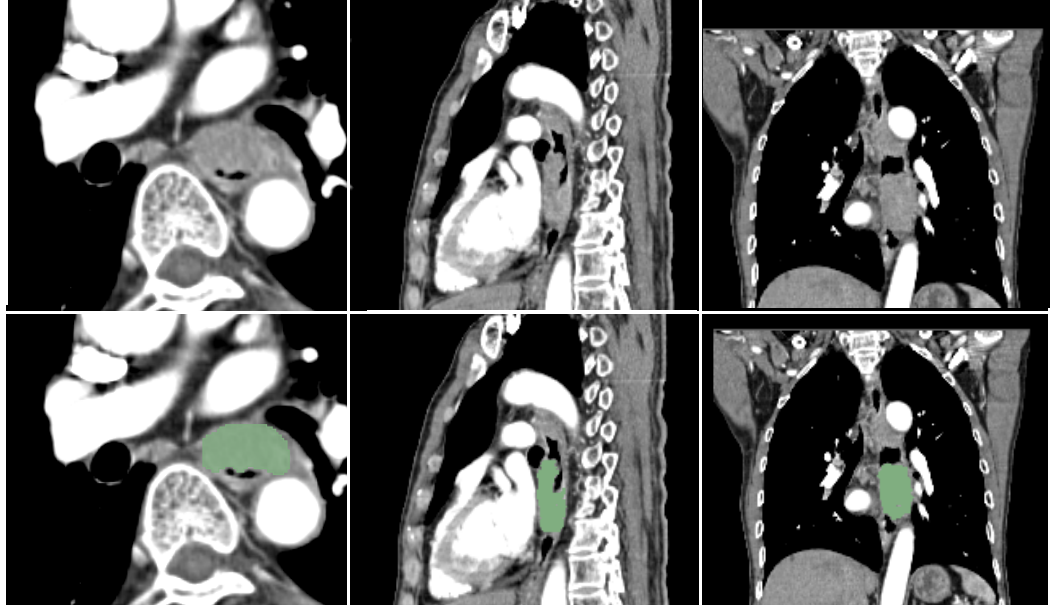


**F**

**E**

**D**

**d**

**A**

**B**

Supplementary figure 1:The demonstration of our working on the tumor segmentation with 3D slicer. (A), (B), (C) shows original computed tomography (CT) images in axial, sagittal, coronal planes;(D), (E), (F) screenshots of segmentation results in axial, sagittal, coronal planes
